# Acetabular dysplasia and the risk of developing hip osteoarthritis within 4–8 years: An individual participant data meta-analysis of 18,807 hips from the World COACH consortium

**DOI:** 10.1016/j.joca.2024.12.001

**Published:** 2024-12-08

**Authors:** Noortje S. Riedstra, Fleur Boel, Michiel M.A. van Buuren, Harbeer Ahedi, Vahid Arbabi, Nigel Arden, Sara J. Baart, Sita M.A. Bierma-Zeinstra, Flavia M. Cicuttini, Timothy F. Cootes, Kay M. Crossley, David T. Felson, Willem Paul Giellis, Joshua Heerey, Graeme Jones, Stefan Kluzek, Nancy E. Lane, Claudia Lindner, John A. Lynch, Joyce B.J. van Meurs, Andrea Mosler, Amanda E. Nelson, Michael C. Nevitt, Edwin H. Oei, Jos Runhaar, Jinchi Tang, Harrie Weinans, Rintje Agricola

**Affiliations:** # Erasmus Medical Center, the Netherlands; † University of Tasmania, Sydney Orthopaedic Research Institute, Australia; ‡ University Medical Center Utrecht, Orthopaedic-Biomechanics Research Group, the Netherlands; § University of Oxford, UK; ¶ Monash University, Australia; ∥ University of Manchester, UK; ## La Trobe University, Australia; †† Boston University School of Medicine, USA; ‡‡ University Medical Center Utrecht, the Netherlands; §§ Menzies Institute for Medical Research, Australia; ¶¶ University of Oxford, UK; ∥∥ University of Nottingham, UK; ### University of California at Davis, United States; ††† University of California, United States; ‡‡‡ University of North Carolina at Chapel Hill, United States; §§§ University Medical Center Utrecht, Delft University of Technology, the Netherlands

**Keywords:** Hip imaging, Risk factor, Developmental dysplasia, Harmonized, Epidemiology

## Abstract

**Objective:**

To study the association between various radiographic definitions of acetabular dysplasia (AD) and incident radiographic hip osteoarthritis (RHOA), and to analyze in subgroups.

**Methods:**

Hips free of RHOA at baseline and with follow-up within 4–8 years were drawn from the World COACH consortium. The Wiberg center edge angle (WCEA), acetabular depth width ratio (ADR), and the modified acetabular index (mAI) were calculated. AD was defined as WCEA≤25°, and for secondary analyses as WCEA≤20°, ADR ≤250, mAI ≥ 13°, and a combination. A logistic regression model with generalized mixed effects with 3 levels adjusted for age, biological sex, and body mass index (BMI) was used. Descriptive statistics stratified by age, biological sex and BMI were reported.

**Results:**

A total of 18,807 hips from 9 studies were included. Baseline characteristics: age 61.84 (± 8.32) years, BMI 27.40 (± 4.49) kg/m^2^, 70.1% women. 4766 hips (25.3%) had WCEA≤25°. Within 4–8 years (mean 5.8 ± 1.6) follow-up, 378 hips (2.0%) developed incident RHOA. We found an association between AD and RHOA (adjusted OR [aOR] 1.80 95% confidence interval [CI] 1.40–2.34). In secondary analyses, all other definitions of AD were also associated with incident RHOA (aOR ranging from 1.52 95% CI 1.19–1.94 to 1.96 95% CI 1.26–3.02). Descriptive statistics showed that the relative risk (RR) in AD hips to develop RHOA was higher compared to non-AD hips in age group 61–70 (RR 1.70), BMI < 25 (RR 1.66), and in female hips (RR 1.73).

**Conclusion:**

AD was consistently associated with incident RHOA. Explorative analyses show that AD hips in women and age group 61–70 years seem to be more at risk of developing RHOA compared to non-AD hips.

## Introduction

There is no curative nonsurgical treatment available for hip osteoarthritis (OA).^1,2^ Therefore, prevention is critical, but there is a lack of knowledge on risk factors for the development of radiographic hip OA (RHOA). Identifying risk factors for this disease should be prioritized.

Subtle features of hip shape may predate the development of OA by many years and might therefore be a preventative target.^3,4^ Acetabular dysplasia (AD) has previously been identified as a risk factor for developing RHOA.^5,6^ AD is defined by insufficient coverage of the femoral head by the acetabulum.^7^ Concentrated focal stress on a relatively small area of the acetabulum^7^ is thought to lead to early mechanical failure of the cartilage, and to eventually cause hip OA.^6,8–10^

A systematic review on hip morphology and OA found an association between AD and RHOA odds ratio [OR] 2.38, 95% confidence interval [CI]: 1.84 to 3.07).^9^ However, when analyzing individual studies, these have yielded conflicting results and highlight the need for robust analysis, avoiding inconsistencies in measurements and definitions.^8,9,11–13^ Single cohorts are likely to be underpowered to determine whether specific high-risk subgroups are responsible for the associations found.^9^ Likely due to the overall low number of included individuals and therefore decreased statistical power, existing prospective cohort studies include hips free of RHOA as well as those with doubtful RHOA at baseline, which may bias the presently known associations. Hips with doubtful RHOA already show mild radiographic changes (possible joint space narrowing [JSN] and signs of osteophytes), which may influence the radiographic measures of AD and represent the first signs of potential osteoarthritic changes.^8,11,13^

Using an individual participant data (IPD) meta-analysis, we aimed to investigate the association between AD, defined by the Wiberg center edge angle (WCEA) ≤25° at baseline, and developing incident RHOA within 4–8 years follow-up. For secondary analyses, we investigated whether other measures of AD and other threshold values to quantify AD were associated with incident RHOA. Finally, we performed subgroup analyses stratified by age, biological sex, and body mass index (BMI) to assess potential high-risk subgroups.

## Methods

### Study design and participants

Participants were drawn from the Worldwide Collaboration on OsteoArthritis prediCtion for the Hip (World COACH) consortium. The World COACH consortium is an international collaboration of all worldwide available prospective cohort studies with sequential pelvic or hip imaging. The consortium profile has previously been published in detail elsewhere.^14^

For the present study, we included all cohorts with a follow-up anteroposterior (AP) pelvic radiograph within 4–8 years of a baseline radiograph, that also had an RHOA score available. This led to the inclusion of 9 cohorts (Cohort Hip and Cohort Knee [CHECK], Multi-center Osteoarthritis Study [MOST], Osteo Arthritis Initiative [OAI], Rotterdam Study-I [RS-I], Rotterdam Study-II [RS-II], Rotterdam Study-III [RS-III], the Chingford Study, The Johnston County Project [JoCo] and the Study of Osteoporotic Fractures [SOF]), and exclusion of two cohorts (Tasmanian Older Adults Cohort [TASOAC] and Femoroacetabular impingement and hip osteoarthritis cohort [FORCe]).

We included hips with known BMI, biological sex, and age at baseline. Next, we excluded hips without an original baseline RHOA score. We then excluded radiographs of insufficient quality for automated AD measurement calculation and all AP hip radiographs as they did not allow for constructing a horizontal reference line to adjust for pelvic rotation. Next, we included only the hips with an original RHOA score at follow-up and excluded all baseline hips with pincer morphology (acetabular overcoverage) as determined by a lateral center edge angle (LCEA) ≥40°. We chose to do the latter to compare hips with AD to a reference group with normal acetabular coverage, and because studies have found an association between pincer morphology and RHOA or total hip replacement (THR).^9,11^ Finally, we included only hips free of any signs of RHOA at baseline (any score=0). Studying a population completely free of RHOA at baseline allows for the determination of true predictors of RHOA, as existing osteophytes may affect the measurement of AD and bias the association between AD and incident RHOA. Furthermore, excluding hips with doubtful RHOA isolates the effect of AD on incidence RHOA rather than the effect of AD on progression in hips that likely already have some form of RHOA. This led to a total inclusion of 18,807 hips.

### Radiographs

AP pelvic radiographs were taken at baseline and at follow-up between 4–8 years in each included cohort, according to cohort-specific protocols which have been published previously^15–19^ (Supplementary material 1). To study the impact of the full-limb films from the MOST cohort on the associations between AD and RHOA, that are otherwise studied on pelvic radiographs, sensitivity analyses were performed excluding hips from the MOST cohort from the primary analysis.

### Radiographic measurements

To avoid measurement variability across cohorts, for the present study all AD measurements were calculated uniformly on baseline radiographs. The bony outline of the proximal femur and acetabulum were automatically annotated on the AP pelvic radiographs with a point set using the BoneFinder^®^ software (www.bone-finder. com; The University of Manchester, UK).^20^ This point set was used to perform automated radiographic measurements using a previously published Python script, which was adapted and validated for World COACH data, for which a detailed description can be found elsewhere.^21,22^ The average of two trained reader’s manual measurements were compared to automated morphological measurements. The average of the trained manual readers was considered the gold standard to which the automated method is compared. Intermethod interclass correlation coefficients (ICCs) range from 0.80 (0.60 – 0.90) for the acetabular depth width ratio (ADR) to 0.88 (0.70 – 0.95) for the WCEA.^23^

**Fig. 1:**
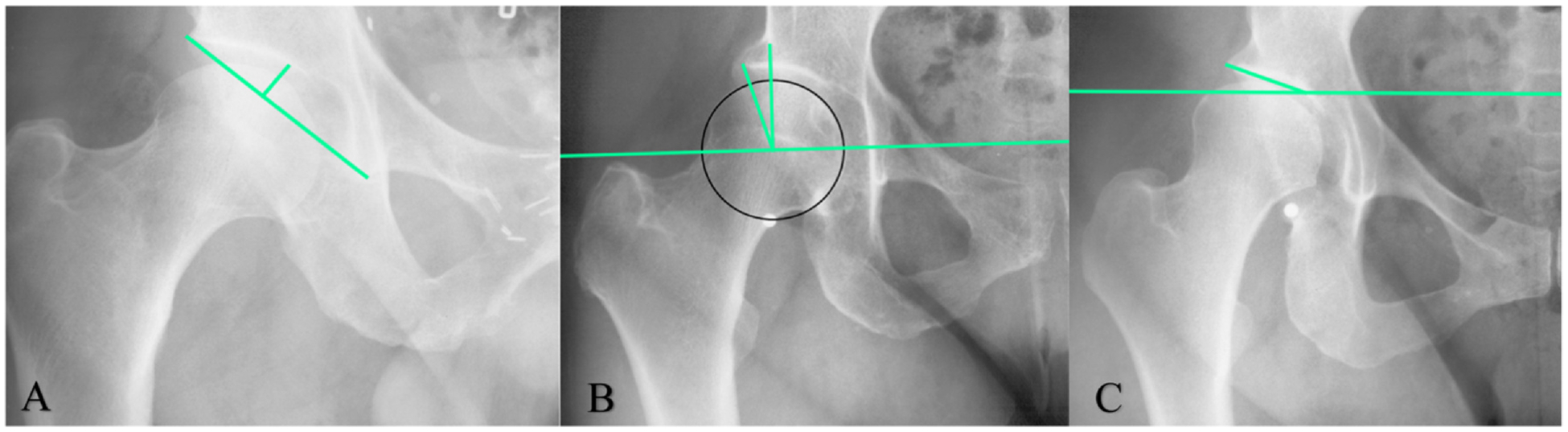
Anteroposterior in pelvic radiographs with three radiographic measurements to define AD. **A: The acetabular depth-width ratio (ADR)**: The acetabular width was defined as a line across the length of the acetabular opening, extending from the lateral edge of the acetabulum to the pelvic teardrop. Next, the acetabular depth was determined by constructing a line perpendicular to the acetabular width, extending from the most medial point of the sourcil. The ADR is defined as the ratio of the depth to the width, multiplied by 1000. **B: The Center Edge Angle of Wiberg (WCEA)**: To determine the center of the femoral head, a best-fitting circle is outlined around the femoral head based on the SSM points. The WCEA is then formed by a line drawn vertically through the center of the femoral head, perpendicular to the horizontal reference line, the second is drawn from the center of the femoral head to the most lateral weight-bearing part of the sourcil. **C: The modified acetabular index (mAI)**: The mAI measures the acetabular roof inclination. The measure is modified, as the original acetabular index is applied to hips with an open triradiate cartilage. The mAI measures inclination from the medial sourcil to the lateral bony part of the acetabulum. **Horizontal reference line** (B +C): To correct for pelvic rotation, a horizontal reference line is calculated based on the average of 4 lines, between 1) both femoral head centers, 2) the most cranial points of the foramen obturator, 3) the most caudal points of the ischial tuberosity and 4) the most caudal points of the pelvic teardrop.

Radiographic measurements to define AD are depicted (Fig. 1). The amount of weight-bearing coverage of the femoral head by the acetabulum is measured by the WCEA^9,11,24^. AD was defined as a WCEA ≤ 25° in the primary analysis and in subgroup analyses, and additionally by WCEA≤20° for secondary analyses.^8,12^ The ADR is a measure of depth of the acetabulum. AD was defined as an ADR ≤ 250 for secondary analyses.^25^ The modified acetabular index (mAI) measures the inclination of the acetabular roof. AD was defined as an mAI ≥ 13° for secondary analyses.^25^ In secondary analyses, the radiographic definitions of AD were studied individually and combined.

### Radiographic Hip Osteoarthritis Grading

At baseline and follow-ups, all included radiographs had scores available by either the Kellgren and Lawrence (KL) classification (CHECK, Chingford, JoCo, MOST, RS-I, RS-II, RS-III),^26^ the modified Croft classification (SOF),^27^ or a modified OA score (OAI).^28^

The KL grading system defines OA severity in five grades(0−4) using a combination of osteophyte, JSN severity, sclerosis and bone deformity.^26^ The modified Croft grading system defines OA severity in five grades(0−4) and is based on 5 radiographic features: JSN, osteophytes, subchondral sclerosis, cyst formation, and deformity.^29^ The modified OA grades are based on the modified Croft grades and defines OA in 3 grades(0−2), where 0 marks hips free of RHOA, 1 defines doubtful RHOA and 2 is definite RHOA.^28^

Original OA scores per cohort were defined as “free of RHOA” (any score 0), “doubtful RHOA” (any score 1), or “definite RHOA” (KL ≥2, Modified Croft ≥2, Modified OA=2, or THR).^8,30,31^

### Outcome measurements

The outcome was incident score “definite RHOA” within 4–8 years follow-up. Additionally for secondary analyses RHOA was defined as an ordinal outcome “free of RHOA”, “doubtful RHOA” and “definite RHOA”.

### Statistical analysis

**Fig. 2:**
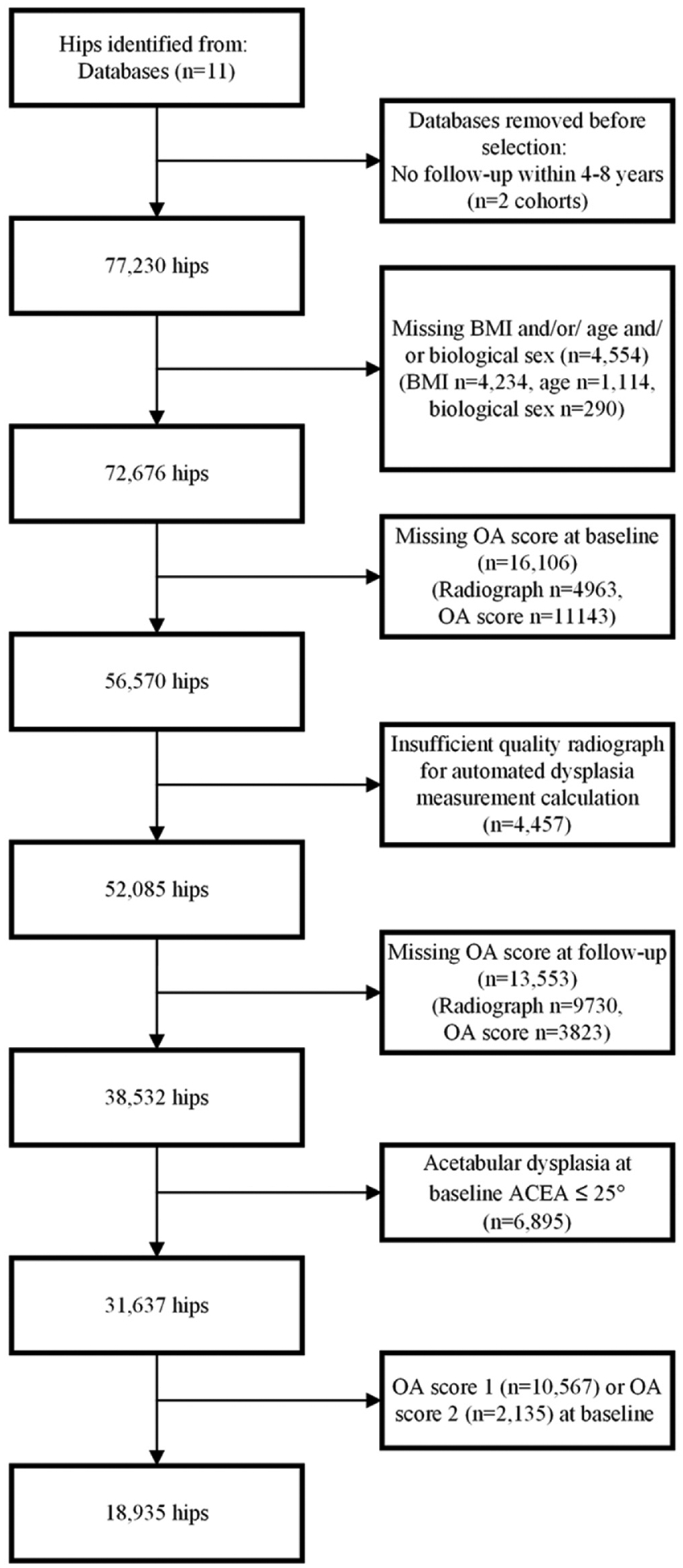
Flow of hips from consortium inclusion to final study population. OA: osteoarthritis. LCEA: lateral center edge angle. AP: anteroposterior. BMI: body mass index.

All statistical analyses were performed in R version 4.1.1. Univariate differences in baseline characteristics between complete included and excluded cases were inspected. This means that we compared the included hips to the hips that were excluded because of OA score of 1 or 2 at baseline (Fig. 2). The associations between baseline AD, defined by the WCEA≤ 25°, and incident RHOA were estimated with mixed effects logistic regression models. Mixed effects were added to account for the potential clustering in the data within cohorts and participants. Random intercepts were determined on both participant and cohort level, with participants nested within the cohorts. The cohort was added as a level in this multi-level model to adjust for possible residual confounding by study differences. An example is the difference between an open population cohort (Chingford, JoCo, RS-I, RS-II, RS-III), and closed population cohort (CHECK, OAI, MOST, SOF). The results are expressed as adjusted OR (aOR) and unadjusted OR with 95% CI and were adjusted for baseline age, sex, and BMI. Additionally, a mixed model for ordinal data, namely RHOA classified as “free of RHOA”, “doubtful RHOA”, and “definite RHOA” was created using a forward build continuation ratio model to assess the impact of doubtful RHOA. Random effects were added to adjust for clustering, and the model was adjusted for baseline age, sex, and BMI. The ordinality assumption of the continuous ratio model was relaxed for AD, allowing the effect of AD to be different for each level of the outcome RHOA at follow-up. The results were presented as an effect plot of the marginal probabilities marginalized over the random effects for women, with mean baseline age and BMI and randomly selected left hip side. Secondary analyses were performed using the same model and 5 definitions of AD: 1) WCEA ≤ 20°, 2) ADR ≤ 250, 3) mAI ≥ 13°, 4) three combined measures (WCEA ≤ 25° and ADR ≤ 250 and mAI ≥ 13°), and 5) a pooled definition of any of the three measures (WCEA ≤ 25° or ADR ≤ 250 or mAI ≥ 13°). In the secondary analysis, when using a WCEA≤20° as the predictor, hips with a WCEA > 20° and WCEA≤25° were excluded from the reference group to compare AD hips to a clean population of hips completely free of AD. In the secondary analysis, when using any of the three measures for AD, the reference group contained hips free of AD and hips with only one or two measures of AD. We used descriptive statistics to explore whether specific subgroups may be more at risk to develop RHOA. Because of limited outcomes, it was not possible to perform subgroup analyses using logistic regression. We reported absolute risk (AR) and relative risk (RR) in AD and non-AD hips of developing RHOA stratified by age groups 40–50, 51–60, 61–70 and > 70 years of age, by BMI by studying groups with a BMI > 25 and BMI≤25, and by biological sex. The following packages in R were used: Logistic regression was performed using the lme4-package.^32^ The continuation ratio model was created using the GLMMadaptive package.^33^ The effect plot was created using the ggplot2-package.^34^

## Results

### Participants

The flow of hips from those available in World COACH to the current final study population is depicted (Fig. 2). 18,807 hips free of any signs of RHOA at baseline were included. The mean interval between the baseline and follow-up radiograph across all cohorts was 5.8 ± 1.6 years. Baseline demographic data stratified per cohort are presented in Table I. Our study population was younger than the excluded hips (61.84 ± 8.32 versus 64.56 ± 8.49 years, respectively); all other baseline characteristics and predictors were similar across included and excluded hips.

### Acetabular dysplasia

At baseline, 4766 (25.3%) hips had AD defined by a WCEA ≤25°. 1164 (6.2%).

according to a WCEA ≤20°, 5917 (31.5%) hips had an ADR ≤250 and 397 (2.1%) hips had an mAI ≥ 13°. The overlap between measures is illustrated in supplementary material 2.

### Incident radiographic hip osteoarthritis

378 hips (2.0%) developed incident RHOA within 4–8 years follow-up. The incidence of RHOA at follow-up per cohort were: CHECK: 13.4% Chingford: 8.4%, JoCo: 6.4%, MOST 0.6%, OAI: 0.5%, SOF: 1.7%, RS-I: 0.9%, RS-II: 0.4%, RS-III: 2.2%.

### Primary analysis: association between acetabular dysplasia and radiographic hip osteoarthritis

A significant association (aOR 1.80 (95% CI 1.40–2.34) between AD (WCEA ≤25°) and incident RHOA within 4–8 years was observed. The association remained statistically significant after adjustment for covariates.

**Fig. 3:**
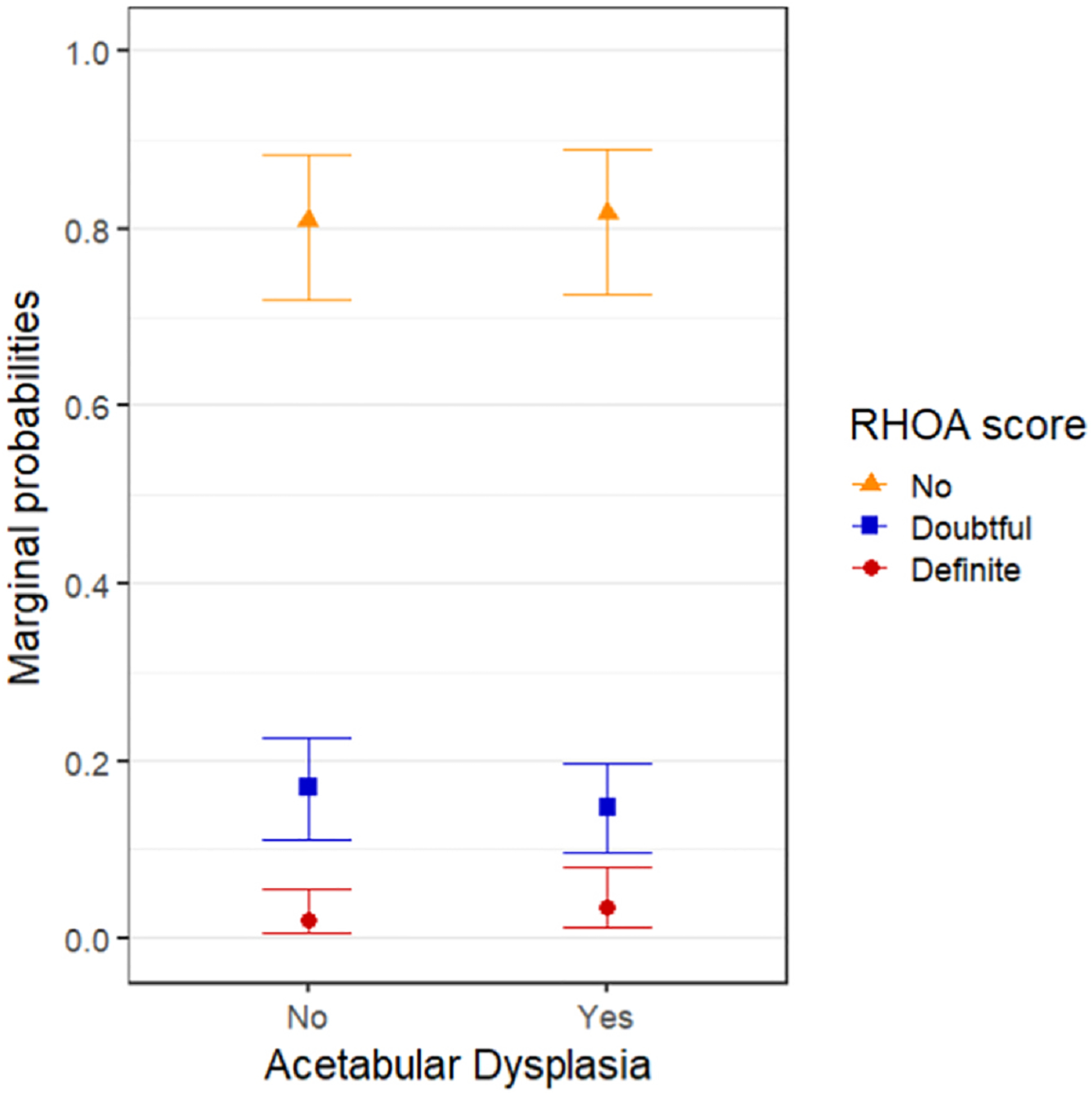
Marginal probabilities of RHOA score within 4–8 years for women aged 62 years and BMI of 27.4 kg/m^2^ in hips with AD (WCEA ≤25°) or without AD. The probabilities were marginalized over the random effects, i.e., cohort and individual and the model was adjusted for age, BMI, biological sex, and hips side.

The effect plot of the marginal probabilities from the mixed model for ordinal data is shown in Fig. 3. All marginal probabilities were calculated for hips free of RHOA, in women aged 62 years with a BMI of 27.4 kg/m^2^ at baseline. The marginal probability for hips with AD to develop doubtful RHOA within 4–8 years is 0.15 (95% CI 0.10–0.20), compared to 0.17 (95% CI 0.11–0.23) for hips free of AD. The marginal probability for AD hips to develop definite RHOA within 4–8 years is 0.03 (95% CI 0.01–0.08), compared to 0.02 (95% CI 0.01–0.06) for AD-free hips.

### Sensitivity analysis excluding MOST

The study population excluding MOST resulted in a total of 17,031 hips. A significant association was found (aOR 1.89 95%CI 1.45 −2.47) between hips with AD (WCEA ≤25°) and incident RHOA in the study population excluding hips from the MOST cohort.

### Secondary analyses: association between various measures of acetabular dysplasia and radiographic hip osteoarthritis

Significant associations between AD defined by WCEA ≤20°, ADR≤250 or either WCEA ≤25° or ADR ≤250 or mAI ≥ 13°) and incident RHOA within 4–8 years were observed. The associations remained statistically significant after adjusting for covariates. Because of a limited number of events (14 hips), it was not possible to calculate the association in the AD defined by mAI≥ 13° group. All ORs are summarized in Table II.

### Subgroup analyses

Descriptive statistics stratified by age group, biological sex, and BMI are summarized in Table III. The RR for hips with AD to develop RHOA was highest in age group 61–70, in hips with BMI < 25, and in women.

## Discussion

This IPD meta-analysis on the association between AD and incident RHOA in a large prospective study of 18,807 hips free of any RHOA at baseline, demonstrated a significant association between AD defined by a WCEA ≤25° and incident RHOA within 4–8 years. Additionally, hips with AD were more likely to progress from being RHOA-free to definite RHOA rather than doubtful RHOA compared to non-AD hips. Secondary analyses showed that other measures of AD (WCEA ≤20°, ADR ≤250 and a combination of WCEA≤25° and ADR ≤250) were also associated with an increased risk of developing RHOA. Descriptive statistics show that AD hips in women, individuals aged 61–70 and individuals with BMI < 25 have a higher RR to develop RHOA.

Several studies have shown that AD is associated with the development of RHOA. The strength of associations in prospective cohort studies ranged from aOR 1.56 (95% CI 1.09–2.24) to aOR 5.45 (95% CI 2.40–12.34).^8,9,11,12,35,36^ Conversely, a number of studies (case-control, prospective and cross-sectional) have failed to find such an association.^13,37,38^ Our results support the finding that AD is associated with RHOA, although the association in the present study is not as strong as previously reported. This may be explained by the fact that the present study population only included hips free of any RHOA at baseline, whereas previous prospective cohort studies also included hips with doubtful RHOA at baseline, in which the stronger associations may reflect an association between AD and progression of RHOA, rather than incident RHOA.^8,9,11,12,35,36^ Furthermore, publication bias may have played a role in selective publication of (strong) associations between AD and RHOA previously, and negative results may have been disfavored.^39^ Time to follow-up as well as how AD and RHOA are measured and defined may also have contributed to variable strengths of associations in prospective studies or absence of an association in cross-sectional studies.

Although generalizable evidence is lacking, it has been hypothesized that AD leads to RHOA only in younger individuals.^11^ Saberi et al. studied hips from RS-I and RS-II with an average age of 65 years at baseline and found that the magnitude of the association AD and RHOA was stronger in persons aged ≤65 years at baseline (OR 2.59 95% CI 1.62–4.16) compared to those aged > 65 years (OR 1.74 95% CI 0.90–3.37). On average, the population in this IPD meta-analysis is younger (61.84 years), which is likely because only hips completely free of RHOA were included at baseline, whereas the aforementioned study also included hips with doubtful RHOA at baseline. Our study lacked sufficient statistical power to stratify associations by age. However, the descriptive subgroup statistics showed that hips with AD aged 61–70 at baseline had an increased RR (1.70 95% CI 1.19–2.44) of developing incident RHOA, which was lower in other age groups although the CI overlaps (age 40–50 RR: 1.07 95% CI 0.53–2.17, age 51–60 RR: 1.40 95% CI 1.02–1.93, age 70+ RR: 1.45 95% CI 0.81–2.61). This finding suggests that younger individuals with AD may not be more at risk, but future studies with sufficient power should further analyze these associations.

The prevalence of AD defined by a WCEA ≤25° in our study population was similar in women (25.8%) compared to men (24.3%) in the study population. Although acetabular undercoverage was equally common in men and women in our study, we found that the RR is significantly lower in men with AD to develop RHOA (RR 0.69 95% CI 0.39–1.23) compared to women (RR 1.73 95% CI 1.37–2.18). It has been hypothesized that women have different joint alignment and thus joint load distribution than men. Estrogen metabolism, or pregnancy related pelvic instability may play a role in sex differences.^40^

We hypothesized that a higher overall body weight may lead to higher joint load and therefore increase the risk of RHOA in overweight individuals with AD. Descriptive statistics show a slightly increased RR for AD hips in individuals who have a BMI < 25 (RR 1.66 95% CI 1.18–2.34) compared to AD hips of individuals with BMI ≥25 (RR 1.42 1.09–1.85) in our study population, but CIs overlap meaning this is likely not significant. Interestingly, a recent study in children found a negative association between being overweight and developing dysplasia.^41^ A previous study in the Rotterdam Study also reported low BMI as a risk factor for AD hips to develop RHOA.^24^

The sensitivity analysis excluding the MOST cohort, as this contained long-limb radiographs rather than AP pelvic radiographs, yielded similar results to the primary analysis. Excluding MOST led to an aOR of 1.89 (95%CI 1.45 −2.47) for the association between AD and RHOA, compared to the primary analysis, which does include the MOST cohort of aOR 1.80 (95% CI 1.40–2.34). By including the hips from the MOST cohort in the primary analysis, we argue that the reported aORs contribute to generalizable results considering the added variation of a different radiographic view. Furthermore, the CIs largely overlap, from which we conclude that there are no statistically important differences between the study population, including and excluding the hips from the MOST cohort.

Quantification of AD may have impacted the previously reported associations between AD and RHOA.^25^ In the present study, WCEA rather than LCEA was employed, as we argue that the weight-bearing surface, rather than the entire bony femoral head coverage, is under stress as a result of AD. Secondly, the threshold to define AD also vary in the literature. We used a threshold of WCEA ≤25° which indicates mild AD and should be kept in mind when interpreting the results. The association between AD when defined by WCEA ≤20° increased, which may indicate that more severe AD increases the risk of developing RHOA. Finally, we found that most studies only use acetabular coverage as a measure of AD, but for the present study we examined if acetabular depth or roof inclination influenced the reported associations. We found that both acetabular undercoverage as well as a shallow acetabulum were significantly associated with RHOA at follow-up in the present population. Whether acetabular roof inclination is also associated with RHOA could not be concluded from the present study, but future studies with long-term follow-up and therefore likely a higher incidence of RHOA may shed light on this measurement as a predictor.

A comprehensive definition of hip OA in epidemiological studies is still lacking.^42^ Commonly used RHOA classification systems are the KL and (modified) Croft grading systems,^26,27,31,42^ for which good ICCs (**κ** = 0.55–0.92) have been reported in the World COACH cohorts.^16,17,26,27,31,42,43^ The inevitable variability in RHOA grading was corrected for in the logistic regression model by accounting for within-cohort effects. The incidence of RHOA in the present study of 2.01% (range per cohort 0.5–13.4%) is relatively low compared to similar studies.^8,11,12,43,44^ Interestingly, the cohorts with the highest incidence of RHOA at follow-up (CHECK (22.6%), JoCo (10.9%) and Chingford (8.6%) were on average younger at baseline than the cohorts with the lowest incidence (OAI (0.5%), RS-II (0.4%) and RS-III (0.2%). The overall low incidence of incident RHOA is likely related to the exclusion of hips with doubtful RHOA at baseline.

The primary strength of the present study is the design. IPD meta-analysis created increased statistical power, reduced publication bias, and allowed for investigation of subgroup effects.^45^ As RHOA is a heterogeneous disease, identifying subgroups for interventions is likely a promising way forward in clinical research. A second benefit of IPD meta-analysis compared to meta-analysis alone is that we were able to choose confounders for all included hips. This allowed us to correct for the same covariates across all cohorts and perform uniform analyses. IPD also helped improve data quality by combining studies with different follow-up and outcomes, to improve generalizability of findings.^46,47^ A second strength is the use of multiple, uniform radiographic measurements to quantify AD. Although the WCEA proved to be the only measure of AD significantly associated with incident RHOA in our analysis, we argue that by additionally studying the ADR and mAI, we captured more of the AD characteristics compared to studies only employing a CEA.^11,35^ A third strength is the uniformity of automated measurements, which removed variability compared to manual measurements and allowed for objective AD measurements across all cohorts.^9^

This study was subject to limitations. The primary limitation is the subjective nature of original OA grading systems, as they rely on subjective assessment of OA features. We accounted for variability in OA scores per cohort in our statistical model and argue that, as these grading systems are still primarily used in a clinical setting, our study represents best current clinical practice. It should also be kept in mind when interpreting the results that RHOA does not equate clinical hip OA.^48^ A second limitation is the variety of radiographic protocols per cohort, such as supine vs. weight-bearing radiographs. However, a recent study showed that for JSN, no difference in measurements between weightbearing or supine AP radiographs was found.^49^ A horizontal reference line allowed for standardization of all other measurements, which reduced variability to a minimum. A third limitation is the lack of statistical power to perform logistic regression in subgroups. As AD has been shown to be a risk factor in younger individuals to develop RHOA, and the number of individuals ≤50 years of age was very limited in the present study.^12^ The current results cannot be generalized to the young adult population (≤50 years). Prospective studies of younger populations are needed to study this further. A limitation of IPD meta-analysis is that it may become prone to selection bias when IPD are only sought for a specific subset of studies. The World COACH cohorts however have been recruited based on a systematic literature search, which has been repeated recently.^50^ Clinicians, researchers, and patients are also actively involved to help identify studies that should be included in the consortium. We therefore argue that publication bias was minimized in our study. We used definitions of AD only in one plane, thereby potentially neglecting anatomical abnormalities that may exist at different planes simultaneously.^51^ We argue however, that by using multiple measurements to define AD, we were still able to capture a wide array of anatomical variability, in line with current clinical practice.

In future studies, identification of modifiable risk factors is essential for prevention of hip OA, as well as improving quality of life by advancing individualized care and identification of new treatments. Hip dysplasia is recognized as a potentially modifiable risk factor. It has been hypothesized that there are two distinct forms of hip dysplasia; developmental dysplasia of the hip (DDH) which is diagnosed during infancy, and AD, which is diagnosed later in life.^52^ A recent study found demographic differences between patients diagnosed with DDH in infancy and adults with AD, supporting this hypothesis.^53^ Examination of newborns for hip instability exemplifies prevention for hip OA in DDH hips, as the plastic hip joint can be stabilized to produce a congruent joint. This study showed that AD in the adult population was highly prevalent depending on the definition used, but the association with RHOA in general may be weaker than previously thought. It is therefore warranted to further our understanding of which individuals with AD specifically are at high risk of developing hip OA, and, assuming that two distinct forms exist, investigate whether one form is clinically more relevant.

In conclusion, this study demonstrated that AD is a risk factor for incident RHOA in hips free of RHOA at baseline. This IPD meta-analysis allowed for a robust analysis of the association between AD and RHOA, due to the large sample size, uniform measurements of AD across all baseline radiographs, and harmonized outcome of RHOA. Identification of modifiable risk factors is essential for prevention of hip OA in the future, as well as improving quality of life by advancing individualized care and identification of new treatments.

## Supplementary Material

Supplementary Material 1

Supplementary Material 2

Supplementary Material 3

Appendix A. Supporting information

Supplementary data associated with this article can be found in the online version at doi:10.1016/j.joca.2024.12.001.

## Figures and Tables

**Table I:** Baseline characteristic of included hips, stratified per cohort.

	CHECK	Ching	JoCo	MOST	OAI	SOF	RS-I	RS-II	RS-III	Total incl.	Total excl.^a^
Hips, n (participants)	753 (457)	727 (464)	614 (442)	1776 (1078)	4552 (2618)	3547 (2399)	2460 (1660)	1810 (1056)	2568 (1463)	18,807 (11,637)	9708 (6772)
Age, mean (sd) years	55.65 (5.24)	53.09 (5.59)	58.21 (8.49)	60.84 (7.43)	59.96 (8.90)	70.07 (4.27)	64.84 (6.46)	62.75 (6.15)	56.13 (4.85)	61.84 (8.32)	64.56 (8.49)
BMI, mean (sd) kg/m2	26.11 (4.02)	25.44 (4.09)	29.62 (6.01)	29.60 (5.03)	28.12 (4.60)	26.42 (4.37)	26.37 (3.53)	27.20 (3.86)	27.46 (4.08)	27.40 (4.49)	27.59 (4.73)
Men, n (%)	117 (15.5)	0(0)	298 (48.5)	501 (28.2)	1895 (41.6)	0 (0.0)	955 (38.8)	756 (41.8)	1109 (43.2)	5631 (29.9)	3158 (32.5)
WCEA ≤20°, n (%)	48 (6.4)	43 (5.9)	18 (2.9)	274 (15.4)	234 (5.1)	106 (3.0)	161 (6.5)	106 (5.9)	174 (6.8)	1164 (6.2)	510 (5.3)
WCEA ≤25°, n (%)	218 (29.07)	173 (23.8)	130 (21.2)	795 (44.8)	1085 (23.8)	575 (16.2)	628 (25.5)	453 (25.0)	709 (27.6)	4766 (25.3)	2105 (21.7)
ADR ≤250, n (%)	248 (32.9)	212 (29.2)	159 (25.9)	715 (40.3)	1273 (28.0)	955 (26.9)	857 (34.8)	651 (36.0)	847 (33.0)	5917 (31.5)	2694 (27.8)
mAI ≥ 13°, n (%)	14 (1.9)	26 (3.6)	11 (1.8)	72 (4.1)	84 (1.8)	54 (1.5)	50 (2.0)	35 (1.9)	51 (2.0)	397 (2.1)	191 (2.0)
OA score=2 follow-up, men/women (%/%)	101 (13.4)	61 (8.4)	39 (6.4)	10 (0.6)	21 (0.5)	60 (1.7)	22 (0.9)	7 (0.4)	57 (2.2)	77/301 (1.4/2.3)	2390 (24.6)

CHECK= Cohort Hip and Cohort Knee, MOST= Multi-center Osteoarthritis Study, OAI= Osteo Arthritis Initiative, RS-I= Rotterdam Study-I, RS-II=Rotterdam Study-II, RS-III= Rotterdam Study-III (RS-III), Ching= the Chingford Study, JoCo=The Johnston County Project, SOF= Study of Osteoporotic Fractures, WCEA= Wiberg Center Edge Angle, ADR= Acetabular Depth-Width Ratio, mAI= Modified Acetabular Index. OA score: 0= no RHOA, 1= Doubtful RHOA, 2= Definite RHOA.

a Excluded hips are defined as all eligible hips for analysis but with OA score 1 or 2 at baseline.

**Table II:** Associations between various radiographic definitions of AD and incident RHOA.

Definition of AD	Hips with AD at baseline, n	Hips with incident RHOA at follow-up, n	Absolute risk (%)	Unadjusted OR (95% CI)^a^	Adjusted OR (95% CI)
WCEA ≤25°	4766	127	127/4766 (2.7)	**1.73 (1.33–2.25)**	**1.80 (1.40–2.34)**
WCEA ≤20°	1164	34	34/1164 (2.9)	**1.80 (1.28–2.52)**	**1.96 (1.26–3.02)**
ADR ≤250	5917	144	144/5917 (2.4)	**1.48 (1.15–1.90)**	**1.53 (1.19–1.96)**
mAI ≥ 13°	397	14	14/397 (3.5)	-^b^	-^b^
WCEA ≤25° & ADR ≤250 & mAI ≥ 13°^c^	351	14	14/351 (4.0)	-^b^	-^b^
WCEA ≤25° or ADR ≤250 or mAI ≥ 13°^d^	7480	176	176/7480 (2.4)	**1.47 (1.16–1.88)**	**1.52 (1.19–1.94)**

ORs were adjusted for age, BMI, biological sex, and hip side, and were accounted for by clustering cohort and individual. WCEA: Wiberg center edge angle. ADR: acetabular depth-width ratio. mAI: modified acetabular index. OR: odds ratio. CI: confidence interval.

Significant associations are printed in **bold**.

a The unadjusted odds ratios are calculated using the logistic regression model with generalized mixed effects with 3 levels (cohort, person and -hip side correlation) unadjusted for age, biological sex, and BMI.

b Too few cases with both predictor and outcome to calculate an OR.

c The reference group contained hips free of AD and hips with only 1 or 2 measures of AD.

d The reference group contained hips free of any measure to define AD.

**Table III:** Absolute and relative risk of hips with acetabular dysplasia (WCEA ≤25°) to develop incident radiographic hip osteoarthritis stratified by age group, BMI, and biological sex.

Strata	Total hips in group, n	Hips with AD (WCEA ≤25°), n	Hips with incident RHOA, n	Hips with AD and incident RHOA, n	Absolute Risk, %^a^	Relative Risk, % (95% CI)^b^
**Age group (years)**						
40–50	1753	526 (30.0)	35 (2.0)	11	0.6	1.07 (0.53–2.17)
51–60	6738	1921 (28.5)	159 (2.4)	57	0.8	1.40 (1.02–1.93)
61–70	7192	1691 (23.5)	128 (1.8)	44	0.6	1.70 (1.19–2.44)
70+	3124	628 (20.1)	56 (1.8)	15	0.5	1.45 (0.81–2.61)
**BMI**						
< 25	5874	1380 (23.5)	142 (2.4)	48	0.8	1.66 (1.18–2.34)
≥25	12,933	3386 (26.2)	236 (1.8)	79	0.6	1.42 (1.09–1.85)
**Biological sex**						
Men	5631	1369 (24.3)	77 (1.4)	14	0.2	0.69 (0.39–1.23)
Women	13,176	3397 (25.8)	301 (2.3)	113	0.9	1.73 (1.37–2.18)

a The absolute risk was calculated using the following equation: (number of hips with pincer morphology and RHOA/Total number of hips in subgroup).

b The relative risk was calculated using the following equation: (number of hips with pincer & RHOA/(number of hips with pincer & RHOA + number of hips with pincer only)) / (number of hips with RHOA without pincer morphology/ (number of hips with RHOA without pincer morphology + number of hips without pincer morphology or RHOA)).

## Data Availability

Data are available upon reasonable request. Data may be obtained from a third party and are not publicly available. We encourage the use of data by third parties, although this is subject to approval by the steering committees of the World COACH consortium and the participating cohorts, as well as to legal boundaries regarding data ownership. A standardized data request form is available for which will be reviewed uniformly in order to consistetenly handle World COACH data requests.
