# Supplementary material for: Acetabular dysplasia and the risk of developing hip osteoarthritis within 4-8 years: An individual participant data meta-analysis of 18,807 hips from the World COACH consortium
Source: Osteoarthritis Cartilage. Author manuscript; Available in PMC 2026 Feb 19. (PMC12239852; doi:10.1016/j.joca.2024.12.001)
Supplement: Supplementary Material [file EMS211780-supplement-Supplementary_Material.zip › 1-s2.0-S1063458424014791-mmc1.docx]

**
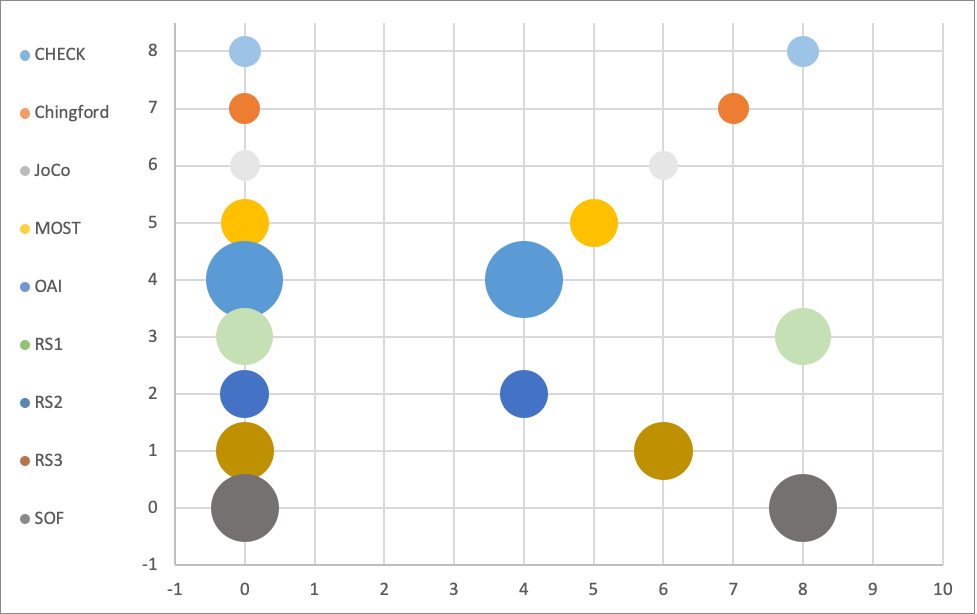
**

**Fig. 1** Radiographs per cohort at baseline and follow-up within 4-8 years. The size of the dot is proportionate to the number of included individuals at baseline and at each follow-up moment.
