# Supplementary material for: Acetabular dysplasia and the risk of developing hip osteoarthritis within 4-8 years: An individual participant data meta-analysis of 18,807 hips from the World COACH consortium
Source: Osteoarthritis Cartilage. Author manuscript; Available in PMC 2026 Feb 19. (PMC12239852; doi:10.1016/j.joca.2024.12.001)
Supplement: Supplementary Material [file EMS211780-supplement-Supplementary_Material.zip › 1-s2.0-S1063458424014791-mmc3.pdf]

Supplementary material 3: absolute and relative risk stratified by biological sex.

Table 1. Absolute and relative risk of hips with acetabular dysplasia to develop incident radiographic hip osteoarthritis in males.

Definition of AD	Total hips in group, n	Hips with AD, n (prevalence, %, 95% CI)	Hips with incident RHOA, n (prevalence, %, 95% CI)	Hips with AD and incident RHOA, n	Absolute Risk, % ***	Relative Risk (95% CI) ****
**WCEA ≤25°**	5,631	1,369 (24.3 (23.2-25.5))	77 (1.4 (1.1-1.7))	14	0.25	0.69 (0.39-1.23)
**WCEA ≤20°**	5,631	300 (5.3 (4.8-5.9))	77 (1.4 (1.1-1.7))	4	0.07	0.97 (0.36-2.65)
**ADR ≤250**	5,631	2,239 (39.8 (38.5-41.1))	77 (1.4 (1.1-1.7))	35	0.62	1.26 (0.81-1.97)
**mAI ≥ 13°**	5,631	105 (1.9 (1.5-2.3))	77 (1.4 (1.1-1.7))	1	0.02	0.69 (0.10-4.93)
**WCEA ≤25° & ADR ≤250 & mAI ≥ 13°***	5,631	100 (1.8 (1.4-2.2)	77 (1.4 (1.1-1.7))	1	0.02	0.73 (0.10-5.18)
**WCEA ≤25° or ADR ≤250 or mAI ≥ 13°****	5,631	2518 (44.7 (43.4-46.0))	77 (1.4 (1.1-1.7))	39	0.69	1.27 (0.81-1.98)

AD: acetabular dysplasia. WCEA: Wiberg center edge angle. ADR: acetabular depth-width ratio. mAI: modified acetabular index. CI: confidence interval.

*The reference group contained hips free of AD and hips with only 1 or 2 measures of AD.

** The reference group contained hips free of any measure to define AD.

***The absolute risk was calculated using the following equation: (number of hips with AD and RHOA/Total number of hips in subgroup)*100%

****The relative risk was calculated using the following equation: (number of hips with AD & RHOA/ (number of hips with AD & RHOA + number of hips with AD only)) / (number of hips with RHOA without AD/ (number of hips with RHOA without AD + number of hips without AD and RHOA))

Table 2. Absolute and relative risk of hips with acetabular dysplasia to develop incident radiographic hip osteoarthritis in females.

Definition of AD	Total hips in group, n	Hips with AD, n (prevalence, %, 95% CI)	Hips with incident RHOA, n (prevalence, %, 95% CI)	Hips with AD and incident RHOA, n	Absolute Risk, % ***	Relative Risk (95% CI) ****
**WCEA ≤25°**	13176	3397 (25.8 (25.0-26.5))	301 (2.3 (2.0-2.6))	113	0.86	1.73 (1.37-2.18)
**WCEA ≤20°**	13176	864 (6.6 (6.1-7.0))	301 (2.3 (2.0-2.6))	30	0.23	1.58 (1.09-2.29)
**ADR ≤250**	13176	3678 (27.9 (27.1-28.7))	301 (2.3 (2.0-2.6))	109	0.83	1.47 (1.16-1.85)
**mAI ≥ 13°**	13176	292 (2.2 (2.0-2.5))	301 (2.3 (2.0-2.6))	13	0.10	1.99 (1.16-3.43)
**WCEA ≤25° & ADR ≤250 & mAI ≥ 13°***	13176	251 (1.9 (1.7-2.2)	301 (2.3 (2.0-2.6))	13	0.10	2.32 (1.35-3.99)
**WCEA ≤25° or ADR ≤250 or mAI ≥ 13°****	13176	4962 (37.7 (36.8-38.5))	301 (2.3 (2.0-2.6))	137	1.04	1.38 (1.11-1.73)

AD: acetabular dysplasia. WCEA: Wiberg center edge angle. ADR: acetabular depth-width ratio. mAI: modified acetabular index. CI: confidence interval.

*The reference group contained hips free of AD and hips with only 1 or 2 measures of AD.

** The reference group contained hips free of any measure to define AD.

***The absolute risk was calculated using the following equation: (number of hips with AD and RHOA/Total number of hips in subgroup)*100%

****The relative risk was calculated using the following equation: (number of hips with AD & RHOA/ (number of hips with AD & RHOA + number of hips with AD only)) / (number of hips with RHOA without AD/ (number of hips with RHOA without AD + number of hips without AD and RHOA))
