# Supplementary material for: Acetabular dysplasia and the risk of developing hip osteoarthritis within 4-8 years: An individual participant data meta-analysis of 18,807 hips from the World COACH consortium
Source: Osteoarthritis Cartilage. Author manuscript; Available in PMC 2026 Feb 19. (PMC12239852; doi:10.1016/j.joca.2024.12.001)
Supplement: Supplementary Material [file EMS211780-supplement-Supplementary_Material.zip › 1-s2.0-S1063458424014791-mmc2.docx]

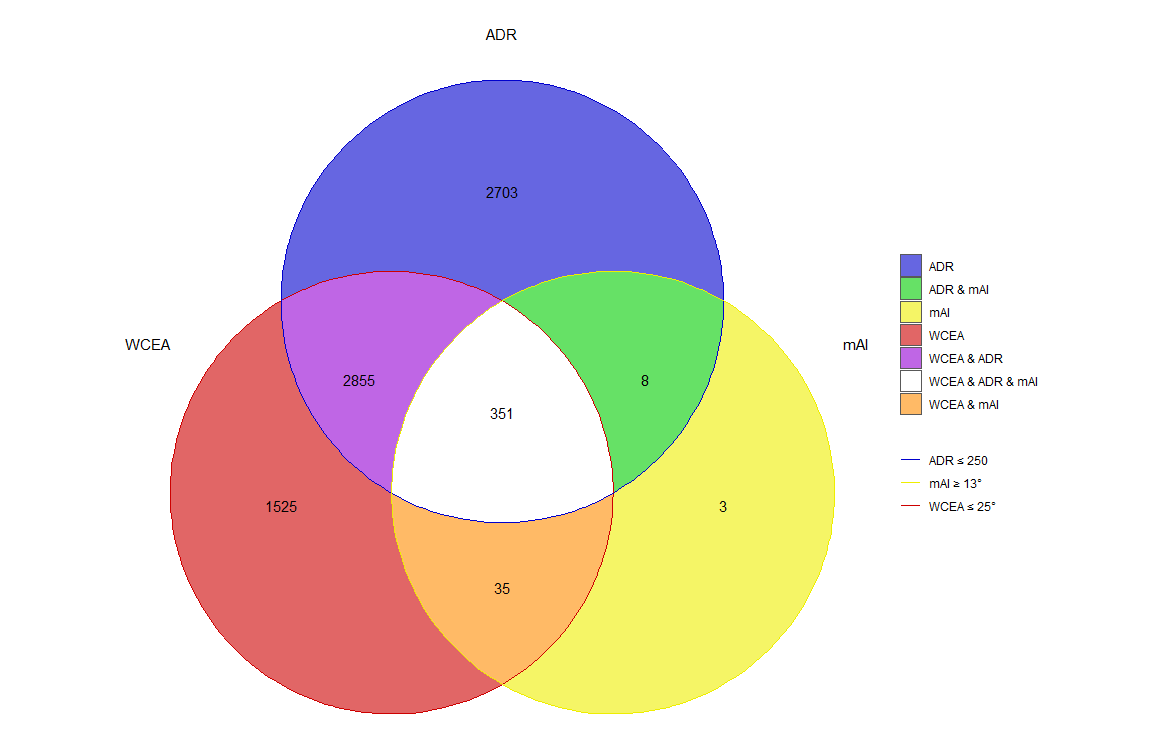


**Fig. 4.** Venn diagram of all AD measures. ADR: acetabular depth-width ratio ≤250. mAI: modified acetabular index ≥13°. WCEA: Wiberg center edge angle ≤25°.
